# Safety Evaluation of Parastar^®^ Plus in Dogs and Assessment of Transferable Residue of Fipronil and Cyphenothrin from Dogs to Humans

**DOI:** 10.3389/fvets.2016.00089

**Published:** 2016-09-30

**Authors:** Katharine M. Case, Natalia M. Vega, Ramesh C. Gupta, Michelle A. Lasher, Terry D. Canerdy

**Affiliations:** ^1^Toxicology Department, Breathitt Veterinary Center, Murray State University, Hopkinsville, KY, USA; ^2^Murray State University, Murray, KY, USA

**Keywords:** parastar^®^ plus, fipronil, cyphenothrin, pyrethroids, ectoparasiticide safety, dogs

## Abstract

Dogs are easily infested with fleas, ticks, and other ectoparasites serving as vectors for transmitting bacterial, viral, and parasitic diseases. Therefore, the use of ectoparasiticides is inevitable and important. The present investigation was undertaken with two specific objectives: one, to evaluate the safety of fipronil and cyphenothrin in dogs after topical application of Parastar^®^ Plus, and two, to determine the transferable residue of these insecticides from dogs to humans. Six healthy, adult dogs (medium length hair, weighing between 20.5 and 27.3 kg) received topical application of Parastar^®^ Plus (2.68 mL; fipronil, 9.8%, and cyphenothrin, 5.2%) on the back between the shoulder blades. At predetermined intervals, dogs were given a full physical exam, and residues of fipronil and cyphenothrin were determined in dog blood and cotton glove extracts using GC/MS. Fipronil and cyphenothrin peaks eluted at 7.453 and 9.913 min, correspondingly, and the compounds were confirmed based on characteristic ions. At no time was fipronil or cyphenothrin residue detected in blood samples. In glove extracts, residues of fipronil and cyphenothrin were maximally present at 24-h posttreatment (43.84 ± 5.69 and 59.26 ± 8.97 ppm, respectively). By 48 h, the residue levels sharply declined (16.89 ± 2.82 and 17.98 ± 2.07 ppm, respectively). The insecticides’ residues were detected in insignificant amounts after 1 week (5.69 ± 2.16 and 10.00 ± 1.51 ppm, respectively), and only in trace amounts after 2 weeks. At no time did any dog show side effects, except itching at the site of Parastar^®^ Plus application. The findings suggest that Parastar^®^ Plus was safe for dogs, and transferable residues of fipronil and cyphenothrin were minimal, posing very little or no health concern to pet owners or veterinary personnel. Of course, veterinary personnel, who handle many dogs daily, may require proper protection to avoid cumulative exposure.

## Introduction

Currently, there are approximately 80 million dogs residing alongside humans in North America. Dogs serve as hosts for several ectoparasites, such as fleas, ticks, lice, and mites. These ectoparasites are capable of spreading many diseases that are significant for both animal and human health. Zoonotic diseases spread by ectoparasites include Lyme disease, plague, ehrlichiosis, Rocky Mountain spotted fever, scabies, and several others. Internal parasites can also be transmitted, such as the flea tapeworm (*Dipylidium caninum*) that frequently infects children (1). Currently, in more than fifteen countries, microcephaly in thousands of babies born to mothers exposed to Zika virus spread by mosquitoes is the most serious human health concern. Because of the close contact between dogs and humans, and the variety of disease vectors for which dogs serve as the host, treatment of ectoparasites is important and inevitable.

**Figure 1:**
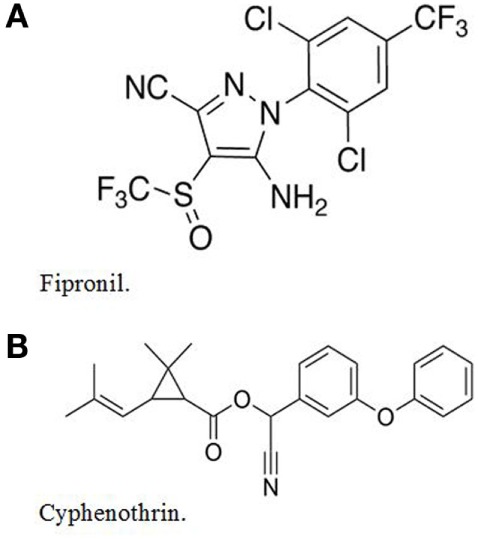
**The chemical structures of (A) fipronil and (B) cyphenothrin**.

Parastar^®^ Plus for Dogs (subsequently referred to as Parastar^®^ Plus) is a monthly topical ectoparasiticide developed by Novartis Animal Health. It kills fleas, several species of ticks, and it controls chewing lice and sarcoptic mange mites (2). The product contains two active ingredients: (1) fipronil: (±)-5-amino-1-[2,6-dichloro-4-(trifluoromethyl)phenyl]-4-[(trifluoromethyl)sulfinyl]-1H-pyrazole-3-carbonitrile, 9.8% and (2) cyphenothrin: [cyano-(3-phenoxyphenyl) methyl] 2,2-dimethyl-3-(2-methylprop-1-enyl)cyclopropane-1-carboxylate, 5.2%. The chemical structures of fipronil and cyphenothrin are shown in Figure 1.

According to the manufacturer, Parastar^®^ Plus begins working within 5 min of application and maintains effectiveness for 30 days (3). It kills fleas (*Ctenocephalides* spp.), four species of ticks (*Dermacentor variabilis, Ixodes* spp., *Amblyomma americanum*, and *Rhipicephalus sanguineus*), mites (*Sarcoptes scabiei* var. *canis*), and lice (order Mallophaga), many of which are vectors for zoonotic diseases of humans.

Fipronil is a phenylpyrazole insecticide that was first developed as an agricultural pesticide in 1987. Due to its lipophilic nature, fipronil is absorbed by the sebaceous glands of the skin, leading its distribution throughout the skin and hair follicles (4, 5), thereby providing ectoparasiticide effect for a month.

Pyrethroids are semisynthetic derivatives of naturally occurring pyrethrins extracted from flowers of the chrysanthemum plant (*Chrysanthemum cinerariaefolium*). Pyrethroids are of two types (type I and type II). Type II pyrethroids are distinguished from type I by having an α-cyano group at the molecule’s alcohol moiety that increases its insecticidal activity. Cyphenothrin is a type II pyrethroid that was first registered at the US Environmental Protection Agency (EPA) in 1991. Like fipronil, cyphenothrin also exhibits lipophilic tendencies that allow it to be distributed among fatty tissue (6) and in oily sebaceous gland secretions.

Safety evaluation of Parastar^®^ Plus (active ingredients, fipronil, and cyphenothrin) in dogs and transferable residues of these insecticides from dogs to humans have not yet been reported. The current investigation was therefore undertaken to evaluate the safety of Parastar^®^ Plus in dogs. Further, since the risk of continuous exposure due to repeated application of Parastar^®^ Plus for children and pet owners exists, the present investigation determined the insecticides (fipronil and cyphenothrin) residues in gloves.

## Materials and Methods

### Animals

Six, privately owned adult dogs (various breeds, with medium length hair coats) weighing between 20.5 and 27.3 kg (45–60 lbs) served as the participants in this investigation, representing both the control (pretreatment) and the experimental (treatment) groups. Each of the dog’s owners signed a consent form detailing the experimental protocol and any risks involved with ectoparasiticide use. Two weeks prior to the beginning of the study, the dogs were taken off any current ectoparasiticides, as well as heartworm prevention. Blood and glove samples were collected on day 0 and were tested for residues of fipronil, cyphenothrin, and any other chemicals. All of the dogs were negative for chemical residues.

### Chemicals

Parastar^®^ Plus was purchased from 1 to 800 PetMeds^®^ (Novartis Animal Health US, Inc., Greensboro, NC, USA). A single application of the product contained 2.68 mL (fipronil 9.8%; cyphenothrin 5.2%) for dogs weighing 45–88 lbs (20.5–40 kg). Analytical grade standards of fipronil and cyphenothrin were purchased from Chem Service, Inc. (West Chester, PA, USA).

### Biohazards

Murray State University’s policy and procedures were followed in this investigation for the disposal of dog blood and gloves.

### Experimental Design

The experimental design of this investigation was based upon previous studies conducted at Murray State University and elsewhere (4, 7–10). In this study, each dog served as its own control. The study protocol was approved by Murray State University’s Institutional Animal Care and Use Committee (IACUC). The IACUC committee approved the use of six dogs based on power analysis.

### Parastar^®^ Plus Application

Parastar^®^ Plus was topically applied in the amount of 2.68 mL directly to the skin between the shoulder blades, according to the manufacturer’s instructions (2).

### Physical Examination

At predetermined intervals, dogs were given a physical examination for the measurement of body weight, heart rate, respiration rate, and body temperature. The dogs were examined for any change in their behavior and the skin was examined for signs of irritation (such as erythema) at the site of Parastar^®^ Plus application. Owners were questioned about their dog’s behavior and whether they observed the dogs itching at the application site.

### Sample Collection

Sampling included blood and topical glove collection. Whole blood (3.5–5.0 mL) was collected from the jugular vein in EDTA tubes on day 0 and at 24, 48, and 72 h, and 1-week post-application of Parastar^®^ Plus. Blood samples were stored in the refrigerator until analysis within 72 h. Topical samples were collected using the wipe sampling technique during which the investigator wore a 100% cotton glove on one hand and pet the dog along its sides and back continuously for 5 min, avoiding the application site. Topical samples were collected on day 0, at 24, 48, and 72 h, and at 1, 2, 3, and 4 weeks post-application of Parastar^®^ Plus. The gloves were then placed into individual (473 mL) glass jars and stored at room temperature until analysis within 72 h.

### Sample Extraction

Blood samples were transferred from EDTA tubes to individual (500 mL) separatory funnels and weighed. Twenty-five milliliter of methylene chloride: petroleum ether (1:1) was added, and the funnels were gently shaken three times and allowed to vent between shakes. The samples sat for 30 min, and then a disposable pipette was used to draw off the solvent and pass it through a sodium sulfate-filled filter into a 50 mL beaker. The solvent was swirled and passed through the filters. A syringe and needle were used to aspirate the remaining 2–3 mL and pass it through a Sep-Pak^®^ cartridge (Waters Corp., Milford, MA, USA) into a clean tube. After it evaporated to dryness, it was reconstituted in an appropriate volume of methylene chloride: petroleum ether for fipronil and cyphenothrin residue analysis by GC/MS.

Gloves were removed from the glass jars, weighed, and then placed into individual 250 mL beakers. Hundred milliliter methylene chloride: petroleum ether (1:1) was added, and the gloves swirled and fully submerged in the solvent, after which they were allowed to sit for 30 min. The solvent was then poured through a sodium sulfate-filled filter into a new 100 mL beaker. During evaporation, the remaining 2–3 mL of solvent were aspirated with a needle and syringe and passed through a Sep-Pak^®^ cartridge into a clean tube. After it evaporated to dryness, it was reconstituted in an appropriate volume of methylene chloride: petroleum ether for fipronil and cyphenothrin residue analysis by GC/MS.

### GC/MS Analysis

**Figure 2:**
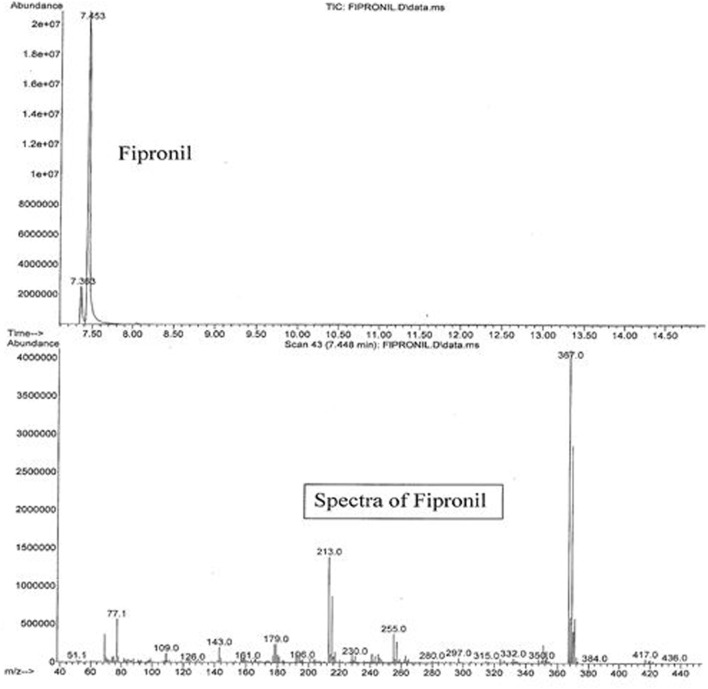
**Total ion chromatogram (upper panel) and ion spectrum (lower panel) of fipronil**.

**Figure 3:**
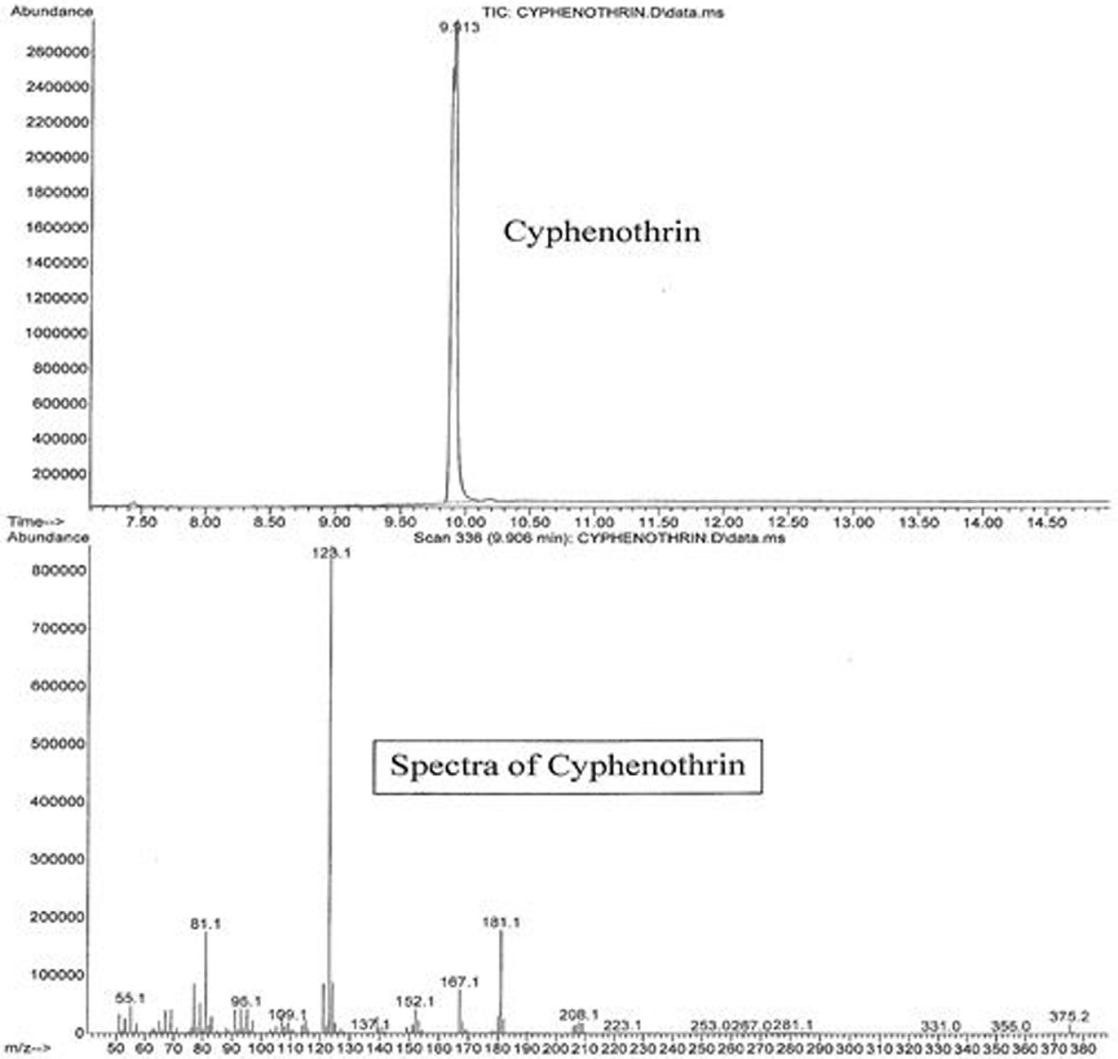
**Total ion chromatogram (upper panel) and ion spectrum (lower panel) of cyphenothrin**.

The active ingredients of Parastar^®^ Plus (fipronil and cyphenothrin) were confirmed and quantified using an agilent gas chromatograph (GC model 7890A)/mass spectrometer (MS model 5975C) coupled with a computer, and their concentrations were expressed in terms of microgram per gram (parts per million). One microliter of the reconstituted extract was injected into the GC. The capillary column used was Ultra II cross-linked with 5% phenyl methyl siloxane coating and was of the following dimensions (25 m × 0.52 μm), which was directly connected to the Mass Selective Detector *via* an interface and heated transfer line. The carrier gas was ultrapure (99.9999%) helium at a flow rate of 2.3 mL/min, and the injector temperature was 200°C. The injector was operated in the splitless mode. A temperature program for the GC-oven was used starting at a temperature of 100°C, and then increased to a final temperature of 300°C in 20°C/min increments. The final temperature was maintained for 5 min. The transfer line temperature was 280°C, and the source temperature was 230°C. The instrument was operated in electron ionization mode, and the ion energy was 70 eV. The total duration of each injection run was 16 min, with a solvent delay of 7 min. Peaks of fipronil and cyphenothrin eluted at 7.453 and 9.913 min, respectively. Sensitivity of the GC/MS for these compounds was in the range of nanogram, and the limit of detection was in the low microgram per gram (parts per million) range. Total ion chromatograms and mass spectra with characteristic ions for fipronil (77.1, 179, 213, 255, 367, 417, and 436) and cyphenothrin (81.1, 123.1, 152.1, 167.1, 181.1, 208.1, and 375.2) are presented in Figures 2 and 3, respectively. The identification and confirmation of each insecticide was based on characteristic ion-based spectrum. Percent recovery for fipronil and cyphenothrin was greater than 95%.

## Results

### Adverse Reactions

At no time during this investigation were adverse reactions to Parastar^®^ Plus observed in any of the dogs. Some participants experienced itching following application, as observed by the owners. It cannot be stated definitively that application of Parastar^®^ Plus caused the clinical signs observed, as patient histories before the study were not entirely known. It was possible that one participant displayed scratching behavior due to an ongoing flea infestation. Flea dirt was observed in this dog’s hair coat throughout the study.

### Fipronil and Cyphenothrin Concentrations in Blood and Glove Samples

The skin and hair coat residue levels of Parastar^®^ Plus’s active ingredients, fipronil (9.8%) and cyphenothrin (5.2%), were expected to reach maximum concentrations at 24-h post-application. In blood, due to slow dermal absorption (11), the maximum concentrations of fipronil and cyphenothrin were expected at 24- to 48-h post-application. Because Parastar^®^ Plus is designed to provide protection for 30 days, insecticide residues on hair coat were expected to be detectable for up to 4 weeks post-application. Residue persistence on hair coat corresponded with these expectations.

**Figure 4:**
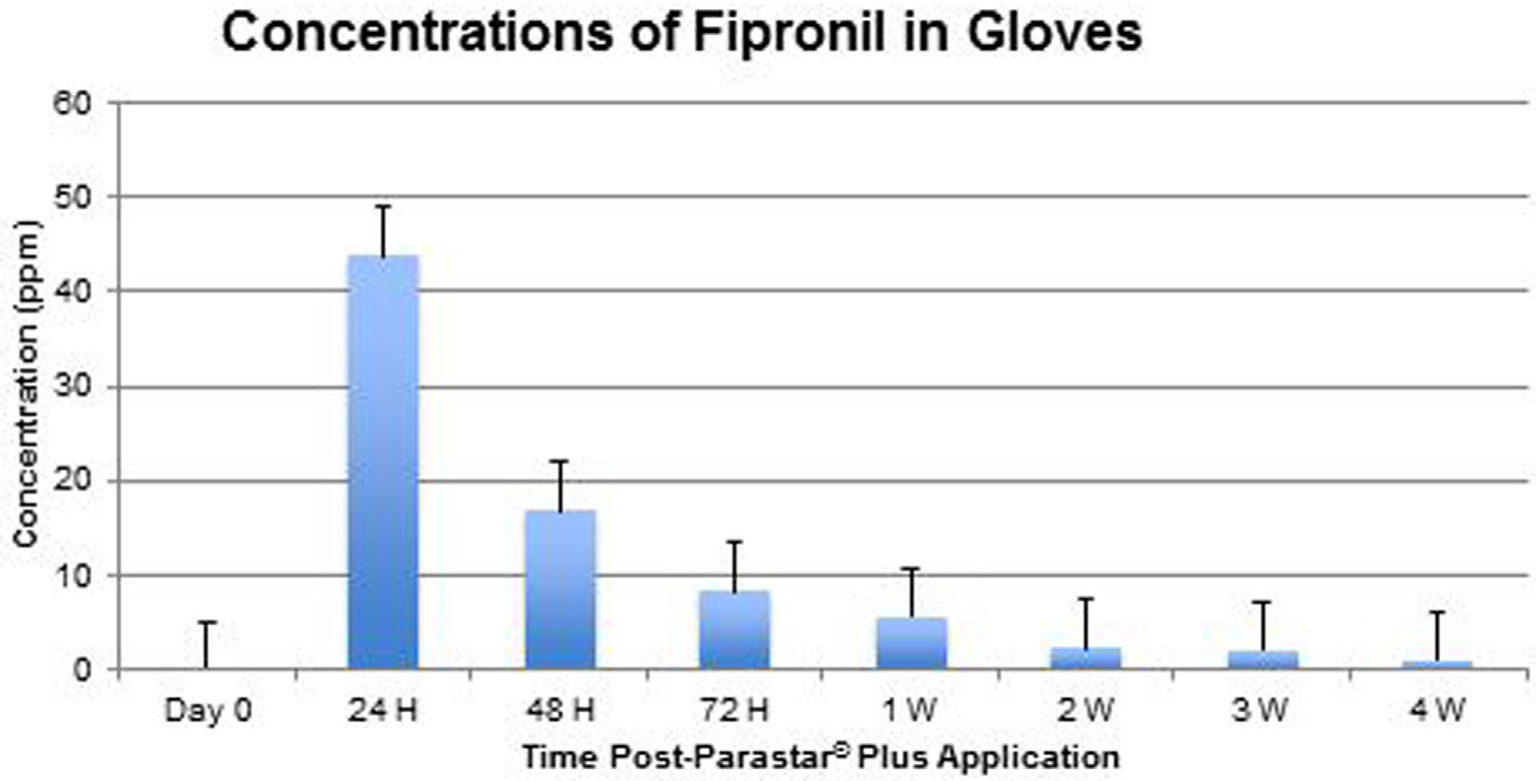
**Concentrations of fipronil in gloves (mean ± SEM)**.

**Figure 5:**
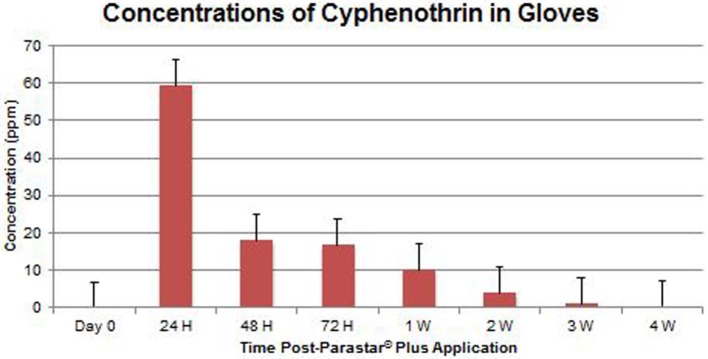
**Concentrations of cyphenothrin in gloves (mean ± SEM)**.

At no time throughout the study duration were fipronil and cyphenothrin detected in blood samples. Data of fipronil and cyphenothrin concentrations in glove extracts are presented in Figures 4 and 5, respectively. Fipronil showed the highest concentration levels in gloves at 24-h post-application with a range of 22.23–59.66 ppm (43.83 ± 5.69 ppm). At the same time point, cyphenothrin concentration was in the range of 26.24–90.40 ppm (59.26 ± 8.97 ppm). At 48-h post-application, residues for both fipronil and cyphenothrin exhibited a steep decline (16.89 ± 2.82 and 17.98 ± 2.07 ppm, respectively) from the 24-h values. At 72 h, the value for fipronil was 8.31 ± 1.69 ppm, and for cyphenothrin it was 16.60 ± 3.19 ppm.

One week after Parastar^®^ Plus application, the value of fipronil was 5.69 ± 2.16 ppm, and of cyphenothrin was 10.00 ± 1.51 ppm. With a decreasing trend at 4 weeks post-application, the value for fipronil was 1.09 ± 0.13 ppm, and for cyphenothrin was 0.43 ± 0.28 ppm.

## Discussion

The present investigation was undertaken with two specific objectives: (1) to evaluate the safety of Parastar^®^ Plus in dogs; and (2) to measure the transferable residue of the active ingredients (fipronil and cyphenothrin) of Parastar^®^ Plus from dog coats to humans. Determining the level of transferable residues of insecticides can aid in assessing the level of exposure and risks to pet owners, veterinary professionals, dog trainers, police, and military K9 units from dogs topically treated with Parastar^®^ Plus.

The results of this study suggest a high level of safety in the use of Parastar^®^ Plus for routine ectoparasite control in dogs, if the manufacturer’s instructions are followed. At no time during this investigation, were adverse reactions to Parastar^®^ Plus observed in any of the dogs. Also, at no time were residues of fipronil and cyphenothrin detected in the dogs’ blood. This indicates that the dermal absorption of fipronil and cyphenothrin is minimal or insignificant, and systemic exposure to these insecticides for dogs is extremely limited. Further, no risk of exposure to insecticides *via* contact with blood exists for veterinary personnel. Residues of fipronil and cyphenothrin on the dogs’ hair coats were found to be maximal at 24 h (43.83 ± 5.69 and 59.26 ± 8.97 ppm, respectively) post-Parastar^®^ Plus application. The manufacturer advises that pet owners limit contact with their dogs during the first 24 h to allow the product to fully dry at the site of application. If this instruction is heeded, the risk of accidental exposure to Parastar^®^ Plus through coat residues is greatly diminished. Of course, local irritation and local reactions could still be observed but have been investigated in this study. This aspect of investigation will be pursued in future studies. Insecticide residues drastically reduced by 48 h from 24 h (fipronil, 61.5% and cyphenothrin, 69.7%). If this 24-h time frame is observed by pet owners, accidental, oral, or dermal exposure to the product would be unlikely, even in children or pet cats.

In the setting of a veterinary clinic, a number of factors can influence the level of insecticide transfer from dogs to veterinary personnel, such as the type of patients seen, the volume of the practice, the number of exposure events occurring per day, and whether members of the staff are observing safety measures like wearing gloves while contacting patients.

In 2009 and 2010, the EPA investigated an increase observed in reports of adverse reactions to spot-on ectoparasiticides from 2007 to 2008. Cyphenothrin, in addition to other pyrethrins/pyrethroids, was among one of the active ingredients highly represented across reports. Small breed dogs and cats were more commonly reported to experience adverse effects to spot-on products (12). The EPA suggested three explanations for why small animals were more susceptible than large animals to the use of these products. One, the weight range for spot-on treatments might vary too greatly. For example, the dose (2.68 mL) of Parastar^®^ Plus used in the current study was for the weight range of 20.5–40 kg. Dogs on the lower end of the range might be receiving twice the dose compared to those at the higher end. Two, owner misuse may be to blame for the increase of adverse events in smaller animals. In an attempt to save money, owners might purchase a dose meant for large dogs and then divide that dose up among multiple small dogs, resulting in a dose that is still too high. In the case of cats, the EPA found a high number of adverse events associated with owners using pyrethrin/pyrethroid-containing products meant for dogs only, despite warnings on the product labels that the treatment was not to be used on cats. Three, small dogs might be more naturally susceptible to the use of spot-on treatments because of biological characteristics specific to the individual animal, even when the correct dose is used. Therefore, it is important for the veterinarian–client–patient relationship to be maintained, as the EPA has suggested that pet owners who purchase spot-on treatments from their veterinarians receive more guidance for their correct usage.

Veterinarians may also be able to aid in choosing an ectoparasiticide that is in fact effective. Some insects are becoming resistant to certain insecticides. This characteristic is referred to as knockdown resistance (*kdr*) and seems to be prevalent in the case of pyrethroids (13). Some species of lice and ticks have already shown to be resistant to pyrethroids, and research suggests that mutations in the genes that form the voltage-gated Na^+^ ion channels are responsible for *kdr*. However, owners might believe that resistance exists when in fact a spot-on treatment does work, especially in the case of fipronil. According to Michael W. Dryden, “It can take time for a flea infestation to be gone, and that has nothing to do with resistance” (14), when speaking of owners claiming that their spot-on treatments that contain fipronil were no longer working. Currently, the practice of using a product having multiple insecticides is common because they provide broad spectrum activity. Ectoparasiticides, such as Parastar^®^ Plus, need to be used as directed by the manufacturer and veterinarians and reapplied at the correct (as indicated earlier) and consistent times (once a month) to obtain the highest efficacy and safety.

Although findings of the current study have shown that the use of Parastar^®^ Plus is safe for both dogs and humans, further investigation is warranted on a larger sample size, dogs of different weight categories, and long-term exposure to Parastar^®^ Plus to observe for any adverse effects. Long-term exposure to Parastar^®^ Plus may result in detectable residue of fipronil and cyphenothrin in dog blood and adverse effects in dogs and greater amounts of residues transferred from dogs to humans. This may require detailed biological monitoring of fipronil and cyphenothrin in dogs as well as humans in our future studies.

Poisonings in dogs and cats due to fipronil or cyphenothrin commonly occur as a result of accidental or intentional ingestion. In humans, poisoning is mainly due to accident or suicide attempt (15–17). In this context, authors are compelled to provide a brief discussion of mechanism of action and toxicity of fipronil and cyphenothrin in this paper. Common clinical signs of fipronil toxicosis are of CNS hyper excitability, including tremors, convulsions, seizures, and death (5). Fipronil is a selective non-competitive blocker of the γ-aminobutyric acid (GABA)-gated chloride channel, manifesting high target specificity between insects versus mammals (18, 19). GABA is an inhibitory neurotransmitter in the insect and mammalian CNS. Following blockage of the GABA-gated chloride channels, hyperexcitation occurs due to abated GABA-induced inhibitory effect, leading to characteristic convulsions, followed by paralysis and death (5). Fipronil-induced clinical signs in humans may include headache, dizziness, fatigue, and itching and irritation of the skin and eyes. Interestingly, a most recent study suggests that a fipronil metabolite fipronil sulfone is more toxic than fipronil itself (20).

Most type I and type II pyrethroids are classified based on the symptoms they produce, but cyphenothrin represents an intermediate pyrethroid. While it is a type II pyrethroid, the symptoms it produces are a combination of whole body tremors (T) and choreoathetosis–salivation (CS) syndrome (21–23). This combination of clinical signs is referred to as TS syndrome. Pyrethroids target the voltage-sensitive sodium (Na^+^) ion channels, causing a depolarization-dependent blockage that holds the channel open for an extended period of time, thereby suppressing the action potential (24, 25). Prolonged opening of the Na^+^ ion channels in the presence of a type II pyrethroid causes type II syndrome or CS syndrome, characterized by burrowing and pawing behaviors, hypersalivation, and ataxia, that progresses to writhing spasms, tonic seizures, and death (22, 24, 26). Type I pyrethroids are recognized for causing a similar but still distinguishable condition referred to as type I or T syndrome. Symptoms of T syndrome are aggressive sparring, clonic seizures, rigor, and death (22).

While both fipronil and cyphenothrin exhibit high target specificity for insects as compared to mammals, due to differences in the CNS, body temperature, and body size (18, 19, 27, 28), safety concerns dealing with accidental poisoning and exposure to humans exist, especially for people who have higher exposure rates to dogs treated with ectoparasiticides (veterinary professionals, dog trainers, military, and police professionals) compared to the general public. Fipronil is classified as a Group C: possible human carcinogen, which, according to the United States EPA (29), includes “agents with limited animal evidence and little or no human data” upon which to establish carcinogenicity. Fipronil’s oral LD_50_ is 97 mg/kg, and its dermal LD_50_ is >2000 mg/kg. Studies strongly suggest that fipronil is an endocrine disruptor (30, 31). Cyphenothrin is grouped into Toxicity Class III: chemicals that represent low toxicity and are labeled with the EPA signal word CAUTION. According to the National Pesticide Information Center (32), chemicals designated under this class are “slightly toxic if eaten, absorbed through the skin, inhaled,” or they cause “moderate eye or skin irritation.” Cyphenothrin’s oral LD_50_ is 318 mg/kg, and its dermal LD_50_ is >5000 mg/kg. These low dermal LD_50_ data suggest that mammalian toxicity *via* skin contact with Parastar^®^ Plus is of low risk in most animals and humans. Biomarkers that could aid in detecting various facets of toxicity of fipronil and pyrethrins/pyrethroids have been described recently by Gupta and Milatovic (33).

### Conclusion and Future Direction

Taken together, findings of this study suggests that if used in accordance with manufacturer’s instructions and allowed 24 h to dry, Parastar^®^ Plus is a safe topical ectoparasiticide and does not pose a threat to the health of dogs or the people who come into contact with them. Since veterinary personnel have direct physical contact with multiple dogs every day, they should observe the proper use of PPE in order to protect themselves against cumulative exposure to Parastar^®^ Plus, as at this time, long-term studies of transferable residues from the combination of fipronil and cyphenothrin have not been completed. In the present investigation, we did not observe eye or skin irritation; in future studies, we will perform histopathology of dermal biopsy at the site of Parastar^®^ Plus application.

## Author Note

Part of the Masters thesis (Katherine M. Case) presented at the 55th Annual Meeting and ToxExpo, New Orleans, LA, USA, March 13–17, 2016. Part of this investigation will also be presented at the 3rd International Veterinary Congress to be held in London, UK, August 18–20, 2016.

## Author Contributions

KC, major contributor; NV, assistant to KC; RG, thesis advisor; ML, data analysis/graphics; and TC, graduate advisor.

## Conflict of Interest Statement

The authors declare that the research was conducted in the absence of any commercial or financial relationships that could be construed as a potential conflict of interest.
